# Optimization of a fresh fecal intraperitoneal injection sepsis model and its divergent dynamics from cecal ligation and puncture in mice

**DOI:** 10.1186/s42826-026-00282-w

**Published:** 2026-05-28

**Authors:** Lixiang Zhao, Zhiwen Wu, Zetian Zhong, Xiaoling Lu, Ruonan Zhang, Li Luo, Yanxin Lu, Xupeng Yue

**Affiliations:** 1 Zhuhai Campus of Zunyi Medical University, Zhuhai, 519000 China; 2 Department of Blood Transfusion, The Fifth Affiliated Hospital of Zunyi Medical University, Zhuhai, 519000 China; 3 Department of Pharmacy, The Fifth Affiliated Hospital of Zunyi Medical University, Zhuhai, 519000 China; 4 Department of Laboratory, The Fifth Affiliated Hospital of Zunyi Medical University, Zhuhai, 519000 China; 5 Department of Pathology, The Fifth Affiliated Hospital of Zunyi Medical University, Zhuhai, 519000 China

**Keywords:** Sepsis, Fresh fecal suspension, Fecal intraperitoneal injection, Cecal ligation and puncture, Animal model

## Abstract

**Background:**

Sepsis remains a critical challenge in intensive care, necessitating reliable animal models that accurately mimic human pathophysiological responses. While cecal ligation and puncture (CLP) is widely considered the gold standard, its inherent variability often limits reproducibility. This study aimed to optimize a fecal intraperitoneal injection (FIP) murine model by evaluating the impact of fecal preparation (fresh vs. lyophilized) and dosage (0.5–1.0 g/kg) on model stability. We systematically compared the optimized FIP model with the conventional CLP method in male BALB/c mice to define their respective pathophysiological characteristics and suitability for therapeutic screening.

**Results:**

Fresh fecal suspensions significantly enhanced model reproducibility compared to dried preparations, which exhibited inconsistent virulence. An optimized FIP dose of 0.7 g/kg induced a hyperacute sepsis phenotype, characterized by rapid systemic bacterial dissemination and severe acute organ damage within 24 h. Crucially, semi-quantitative histological scoring confirmed that FIP triggered a synchronized, hyperacute injury spike across the lung, kidney, liver, and heart, whereas the CLP model exhibited a more protracted, progressive exacerbation of organ dysfunction through 48 h. Hematological analysis further revealed that while both models induced systemic inflammation, the FIP model provided a much sharper and predictable onset of severe leukopenia and multi-organ failure.

**Conclusions:**

The optimized FIP model, characterized by its procedural simplicity, high controllability, and superior reproducibility, serves as a robust platform for investigating the early, fulminant pathophysiological mechanisms of unmitigated sepsis. Conversely, the CLP model remains the preferred choice for studies focusing on protracted infection and chronic organ dysfunction. These findings provide a methodological framework for selecting appropriate sepsis models based on specific research objectives in experimental medicine.

**Clinical trial number:**

Not applicable.

**Supplementary Information:**

The online version contains supplementary material available at 10.1186/s42826-026-00282-w.

## Background

Sepsis is characterized by life-threatening organ dysfunction resulting from a dysregulated host response to infection [1]. This complex clinical syndrome involves intertwined physiological, pathological, and biochemical disturbances. Despite advancements in supportive care, sepsis imposes a substantial global burden, with approximately 48.9 million cases and 11.0 million deaths annually [2]. Effective therapies remain limited, necessitating reliable animal models to elucidate disease mechanisms and translate interventions into clinical practice [3].

Experimental sepsis models primarily include exogenous endotoxin administration (e.g., lipopolysaccharide, LPS), exogenous live pathogen inoculation (e.g., fecal intraperitoneal injection, FIP), and endogenous host barrier disruption (e.g., cecal ligation and puncture, CLP) [4]. While LPS models are highly reproducible, they fail to mimic the polymicrobial nature or the full clinical course of human sepsis [5]. Similarly, CLP is considered the gold standard but suffers from high inter-operator variability and challenges in standardization [6, 7]. In contrast, FIP offers operational simplicity, high standardization potential, and the ability to induce polymicrobial sepsis. However, its implementation is often hindered by a lack of unified protocols for fecal preparation and dosing. Crucially, while the Minimum Quality Threshold in Pre-clinical Sepsis Studies (MQTiPSS) emphasizes the importance of clinical mimicry [8], the foundational reliability of the induction method itself remains the primary bottleneck to achieving these standards.

To address these limitations, the present study systematically optimized the FIP procedure—including fecal processing, filtration, and standardized dosing—to establish a stable protocol. We further conducted a multi-dimensional comparison between this optimized FIP model and the traditional CLP model to provide a rational basis for model selection in sepsis research.

## Methods

### Experimental animals

Specific pathogen-free (SPF) male BALB/c mice (8–10 weeks old; 25–30 g) were purchased from the Guangdong Medical Laboratory Animal Center (license SCXK (Yue) 2020-0051) and housed under previously described conditions [9]. The BALB/c genetic background was specifically selected for this study due to its well-characterized high susceptibility to polymicrobial peritonitis and endotoxic shock, which provides a highly reproducible biological foundation for tracking the rapid and fulminant trajectory of acute sepsis [10, 11]. Briefly, animals were acclimated for one week under SPF conditions, maintained at a constant temperature of 22 ± 2 °C and 50–60% humidity, with a 12-hour light/dark cycle. They were provided with standard laboratory chow and water ad libitum. All experimental procedures and this study were reported in accordance with ARRIVE guidelines. The study was approved by the Ethics Committee for Animal Experiments of Zunyi Medical University (approval no. ZHSC-2-[2024]078). All methods were performed in accordance with the relevant guidelines and regulations. At the designated time points (24, 48, or 72 h) or upon reaching humane endpoints, mice were euthanized by cervical dislocation under deep anesthesia induced by Zoletil 50 (50 mg/kg, i.p.; Virbac, Carros, France) to minimize suffering.

### Establishment of the CLP model

The CLP procedure was performed according to previously described methods [12]. Prior to the surgical procedure, mice were fasted for 12 h and anesthetized with an intraperitoneal injection of Zoletil 50 (50 mg/kg; Virbac, Carros, France). Following a ~ 1 cm midline laparotomy, the distal half of the cecum was ligated with a 4 − 0 suture and punctured twice with a 21-gauge needle. A minimal amount of fecal content was extruded to ensure patency. Following closure, mice received subcutaneous fluid resuscitation (37 °C sterile saline, 5 mL/100 g). Sham-operated mice underwent the same surgical steps excluding ligation and puncture. To minimize inter-operator variability, all procedures were performed by a single investigator.

### Establishment of the optimized FIP model

To minimize variability driven by moisture content and circadian rhythms, donor feces were strictly collected between 9:00 and 11:00 AM. To further minimize inter-individual variability in the baseline gut microbiota, healthy donor mice of the same batch, sex, and age were selected. Donors were placed in empty cages lined with sterile filter paper to collect fresh feces excreted within a strict 30-min window. Fecal pellets that were urine-contaminated, discolored, or desiccated were strictly excluded; only moist, well-formed pellets were retained. Subsequently, all eligible samples were pooled in a sterile dish and gently mixed to achieve a homogenized microbial composition, thereby mitigating specific microbiomic deviations from individual mice. We then compared suspensions derived from oven-dried (37 °C) feces versus fresh feces. Crude suspensions were prepared in sterile saline to target doses of 0.5–0.8 g/kg.

To ensure homogeneity of the inoculum, a two-step filtration process was employed: a 0.5-mm mesh followed by a 70-µm mesh. The filtrates were maintained on ice and utilized within 2 h of preparation to preserve bacterial viability and ensure all injections were performed within a strictly standardized time window, thereby minimizing variability related to circadian rhythms. Mice received intraperitoneal injections at a fixed volume of 10 mL/kg. Control animals (normal saline [NS] group) received an equivalent volume of sterile saline. In this baseline optimization study, additional fluid resuscitation and analgesics were intentionally withheld from the FIP mice to isolate the pathophysiological effects of the bacterial challenge without pharmacological confounders.

### Monitoring and assessment

Sepsis induction was confirmed by clinical manifestations, including piloerection, reduced activity, and labored breathing. Survival was monitored at 12-hour intervals, while disease severity was evaluated using the Murine Sepsis Score (MSS). As detailed in Additional file 1, this scoring system assesses several clinical parameters, such as appearance, level of consciousness, activity, response to stimuli, ocular discharge, and respiratory quality [13, 14].

### Bacterial burden and biochemical analysis

Bacterial loads in whole blood and peritoneal lavage fluid were quantified via colony-forming unit (CFU) counts on blood agar plates (Huankai Microbial, Guangzhou, China) after 24 h of incubation at 37℃. Serum biomarkers, including alanine aminotransferase (ALT), aspartate aminotransferase (AST), blood urea nitrogen (BUN), and serum creatinine (Scr), were measured using a cobas 8000-c701 automated biochemical analyzer (Roche Diagnostics, Basel, Switzerland). Complete blood counts, encompassing white blood cells (WBC), neutrophils (NEU), lymphocytes (LYM), monocytes (MON), and platelets (PLT), were determined using a Mindray BC-7500 series automated hematology analyzer (Mindray, Shenzhen, China).

### Histopathology

Tissues (heart, liver, spleen, lung, kidney, and small intestine) were collected, fixed in 4% paraformaldehyde, paraffin-embedded, and stained with hematoxylin and eosin (H&E; Servicebio, Wuhan, China). Pathological changes were evaluated under light microscopy. For the semi-quantitative assessment of multi-organ injury severity, histological scoring was performed by two independent pathologists blinded to the experimental groups. For each section, at least ten randomly selected high-power fields (× 400) were evaluated.

Heart injury: Evaluated using the Zingarelli scoring system (scale 0–4) based on the presence and severity of interstitial edema, neutrophil infiltration, contraction bands, and myocardial necrosis [15, 16]. Liver injury: Assessed using the Suzuki scoring criteria. A composite score (scale 0–12) was calculated as the sum of three independent parameters graded from 0 to 4: sinusoidal congestion, hepatocyte cytoplasmic vacuolization, and parenchymal necrosis [17, 18]. Lung injury: Quantified according to the official American Thoracic Society (ATS) guidelines (Matute-Bello scoring system). A weighted composite score ranging from 0 to 1 was computed based on five variables: neutrophils in the alveolar space, neutrophils in the interstitial space, hyaline membranes, proteinaceous debris, and alveolar septal thickening [19, 20]. Kidney injury: Evaluated using the Paller scoring system. A total of 100 randomly selected tubules in the corticomedullary junction were assessed per section. Cumulative points were assigned for tubular epithelial flattening, brush border loss, bleb formation, cytoplasmic vacuolization, interstitial edema, and cell sloughing or cast formation [21, 22].

### Statistical analysis

Statistical analyses were performed using SPSS version 29.0 (IBM Corp., Armonk, NY, USA). Normality was assessed prior to hypothesis testing. Survival rates were evaluated using the Log-rank (Mantel-Cox) test. Continuous variables with normal distribution are expressed as mean ± standard deviation (SD) and were compared using one-way ANOVA followed by Dunnett’s post hoc test. Discrete or non-normally distributed data, such as the MSS and semi-quantitative histological scores, are presented as the median with interquartile range (IQR); these were analyzed using the Kruskal-Wallis test with Dunn’s test for post hoc comparisons. A two-sided *P* < 0.05 was considered statistically significant.

## Results

### Impact of fecal state on model reproducibility

**Fig. 1:**
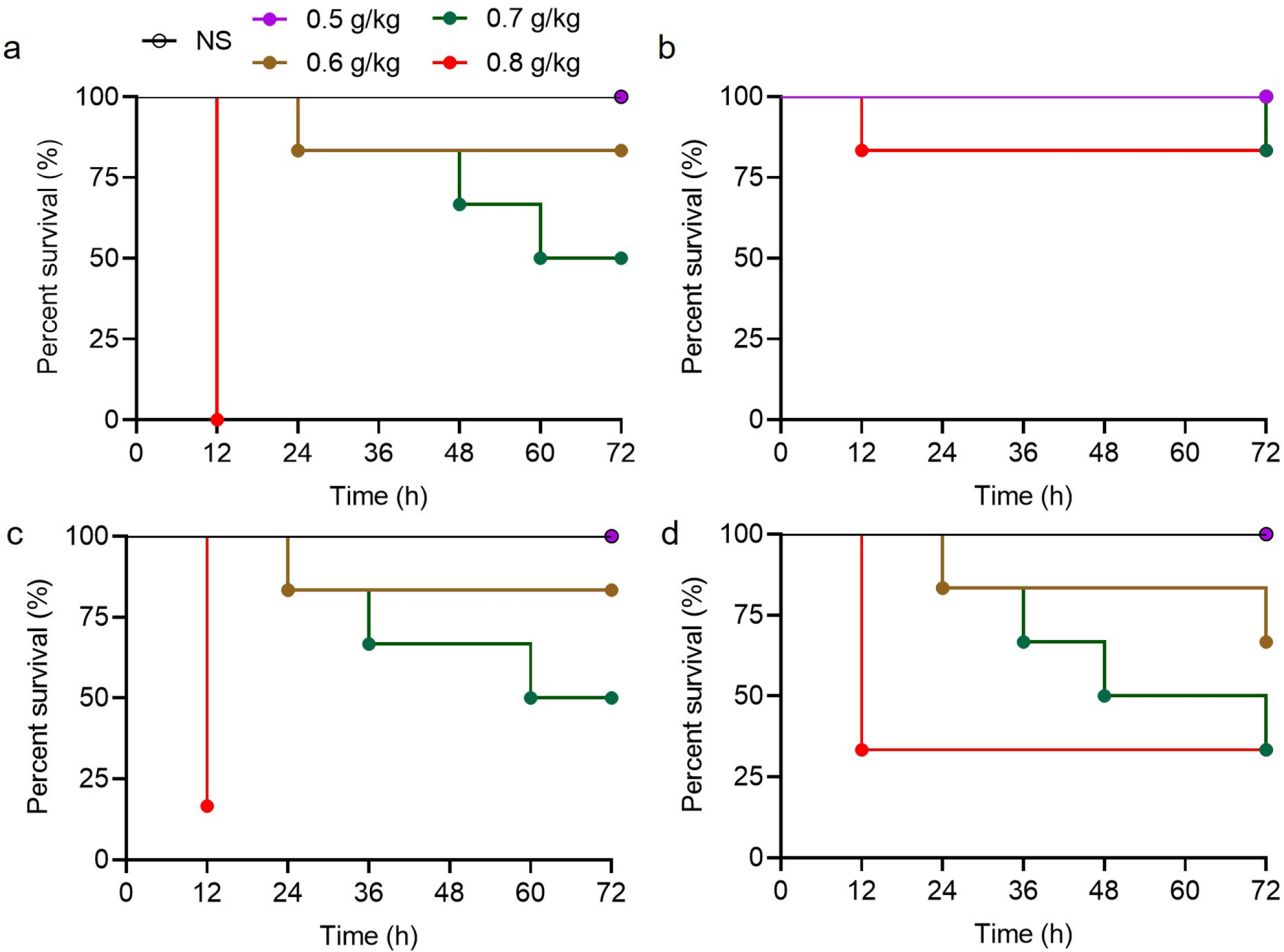
Comparison of model reproducibility between dry and fresh fecal suspensions in the fecal intraperitoneal injection (FIP) model. **a, b** Survival curves from two independent experiments using dry fecal suspension. **c, d** Survival curves from two independent experiments using fresh fecal suspension. Mice were injected at doses of 0.5, 0.6, 0.7, or 0.8 g/kg (*n* = 6 per group). Survival was monitored for 72 h post-injection. NS: normal saline

The standardization of the inoculum substrate is a fundamental prerequisite for ensuring the consistency and physiological relevance of the fecal intraperitoneal injection (FIP) model. To determine the influence of fecal state on reproducibility, survival outcomes were evaluated following the administration of either oven-dried or fresh fecal suspensions. In experiments utilizing dried fecal preparations, survival curves demonstrated substantial variability even at identical doses (0.5–0.8 g/kg) (Fig. 1a, b). This poor reproducibility was likely associated with altered microbial viability and reduced suspension uniformity, which manifested operationally as particle aggregation and frequent syringe clogging. Conversely, suspensions prepared from fresh feces yielded highly consistent survival profiles across independent experimental trials (Fig. 1c, d). Consequently, fresh fecal suspensions were adopted for all subsequent standardized FIP modeling procedures.

### Identification of an optimal FIP dose and comparison with CLP

**Fig. 2:**
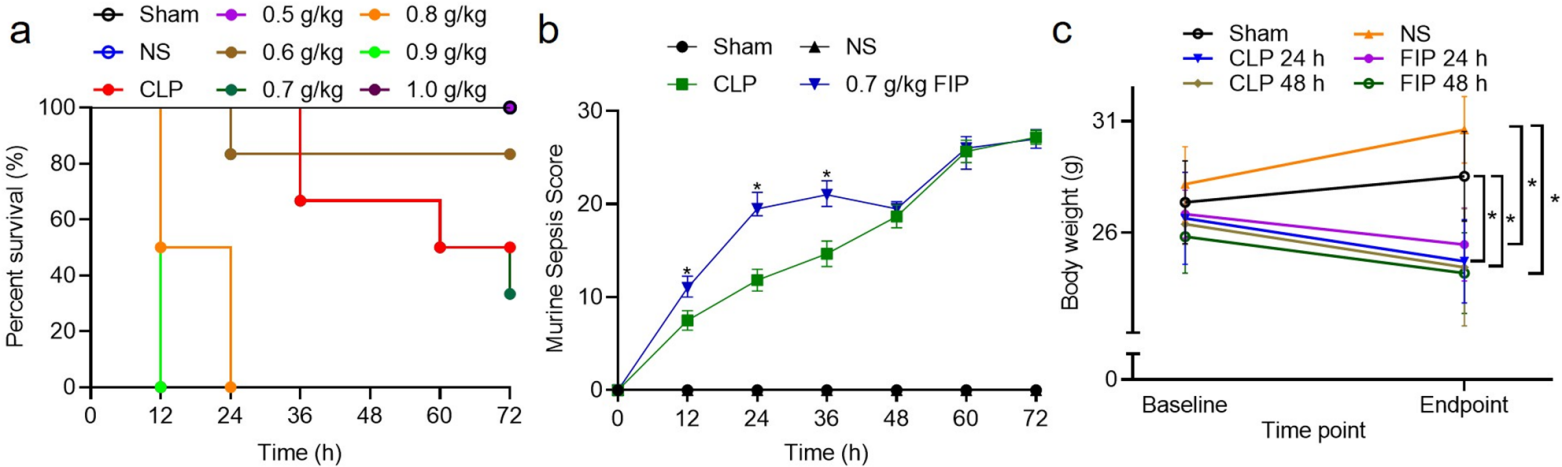
Assessment of survival trajectories, disease severity, and body weight changes between the CLP and optimized fresh FIP models. **a** Survival curves of mice in Sham, CLP, NS, and FIP groups across a dosage range of fresh fecal suspensions (0.5–1.0 g/kg). **b** Dynamic assessment of the Murine Sepsis Score (MSS) at 12-h intervals over 72 h. Data for MSS are presented as median with interquartile range (IQR) and analyzed using the Kruskal-Wallis test followed by Dunn’s post hoc test (*P* < 0.05 vs. CLP group at the same time point). **c** Changes in body weight at 24 and 48 h post-modeling. “Endpoint” refers to the time of euthanasia (24–48 h). Data for body weight are presented as mean ± SD and analyzed by one-way ANOVA. Statistical significance between the indicated groups is highlighted with brackets (*P* < 0.05). CLP: cecal ligation and puncture; FIP: fecal intraperitoneal injection; NS: normal saline. Exact *P* values for all relevant statistical comparisons are detailed in Additional file 2

Establishing a precise dose-response relationship is essential for facilitating meaningful head-to-head comparisons between the optimized FIP model and the established cecal ligation and puncture (CLP) protocol. To identify an optimal induction dose, survival was monitored across a dose range of fresh fecal suspensions (0.5–1.0 g/kg) (Fig. 2a). A clear dose-response relationship was observed; all mice survived at a dose of 0.5 g/kg, whereas complete mortality occurred within 24 h at doses ≥ 0.8 g/kg (Fig. 2a). The survival trajectory of the 0.7 g/kg FIP group most closely resembled that of the CLP group, leading to its selection as the standard dose for comparative evaluation. Monitoring of the murine sepsis score (MSS) revealed that the FIP group consistently exhibited higher disease severity than the CLP group at 12, 24, and 36 h *(P* < 0.05) (Fig. 2b). Furthermore, significant body weight reductions were recorded in the CLP group at 24 and 48 h *(P* < 0.05) and in the 0.7 g/kg FIP group *(P* < 0.05) compared to their respective controls (Fig. 2c).

### Comparison of bacterial loads in blood and peritoneal lavage fluid

**Fig. 3:**
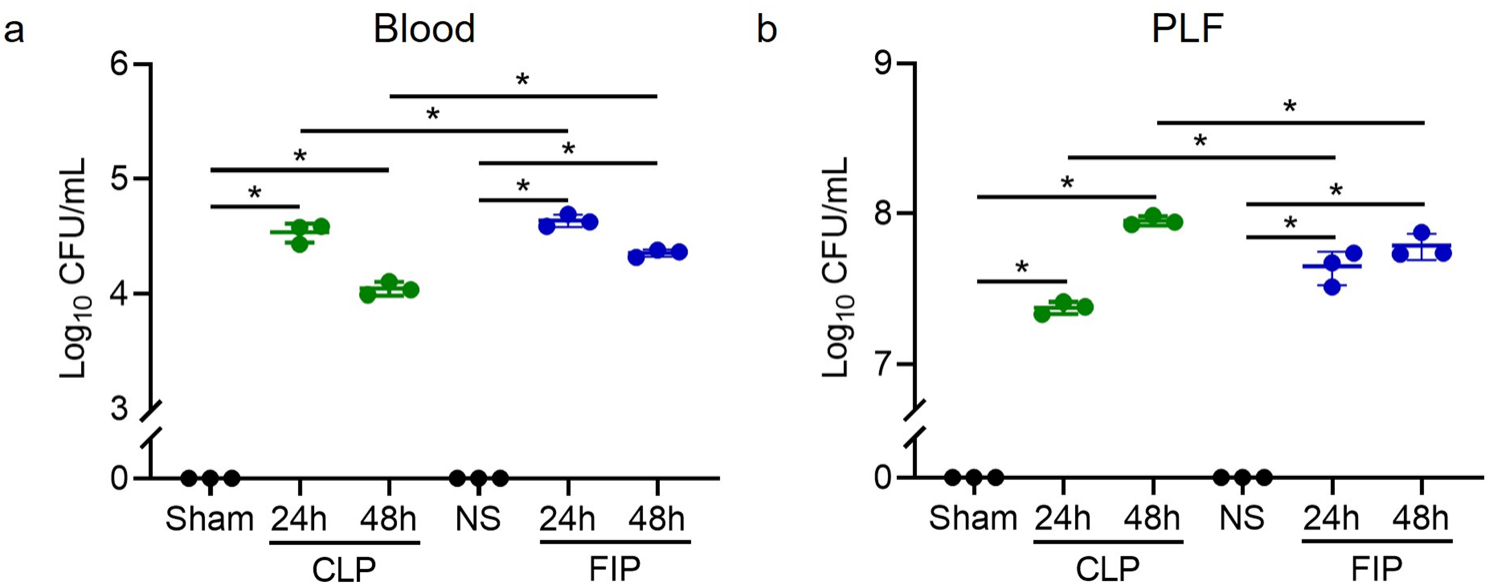
Quantitative bacterial burdens in the systemic circulation and peritoneal cavity at 24 and 48 h post-modeling. **a** Bacterial loads in whole blood. **b** Bacterial loads in the peritoneal lavage fluid (PLF). Data are presented as mean ± SD with individual data points overlaid (*n* = 3). The Y-axes are presented on a log_10_ scale with a broken axis to simultaneously display samples with undetectable bacterial growth (0 CFU) in the control groups and the exponential bacterial proliferation in the septic groups. Statistical significance between the indicated groups is highlighted with brackets (^*^P < 0.05). CLP: cecal ligation and puncture; FIP: fecal intraperitoneal injection; NS: normal saline. Exact *P* values for all relevant statistical comparisons are detailed in Additional file 2

Quantification of bacterial dissemination serves as a direct indicator of infectious severity and the efficacy of host clearance mechanisms across different modeling strategies. Substantial bacterial dissemination was observed in both models, with peritoneal bacterial loads (10^7^–10^8^ CFU/mL) consistently exceeding circulating concentrations. In the blood, bacterial burdens were significantly increased in the CLP group compared with sham controls (*P* < 0.05) and in the FIP group compared with NS controls (*P* < 0.05) at both 24 and 48 h. However, blood bacterial loads were significantly higher in the FIP group than in the CLP group at these same time points *(P* < 0.05) (Fig. 3a). This disparity likely stems from the different infection dynamics of the two models: FIP involves an immediate, high-density bacterial bolus injection, leading to rapid systemic translocation, whereas CLP represents a progressively evolving infection through gradual leakage from the punctured cecum. This characteristic suggests that the optimized FIP model is particularly effective for simulating acute, fulminant sepsis. Furthermore, peritoneal lavage bacterial loads were significantly elevated in the CLP group relative to sham controls (*P* < 0.05) and in the FIP group relative to NS controls *(P* < 0.05) (Fig. 3b). Comparison between the models indicated that peritoneal bacterial loads were significantly higher in the FIP group than in the CLP group at 24 h *(P* < 0.05) but became significantly lower by 48 h *(P* < 0.05) (Fig. 3b). This crossover likely reflects the host’s immune clearance of the initial FIP bolus, contrasted with the persistent microbial influx from the unsealed cecal leak in the CLP model, which serves as a continuous source of infection.

### Systemic inflammatory response and organ dysfunction

The characterization of hematological and biochemical markers is necessary to validate systemic injury and to delineate the extent of multi-organ failure induced by sepsis. To this end, hematological parameters, including WBC, NEU, LYM, and MON counts, were analyzed to evaluate systemic inflammation and immune status, while serum biochemical markers, specifically ALT and AST for hepatic function and BUN and Scr for renal function, were measured to assess organ-specific injury.

**Fig. 4:**
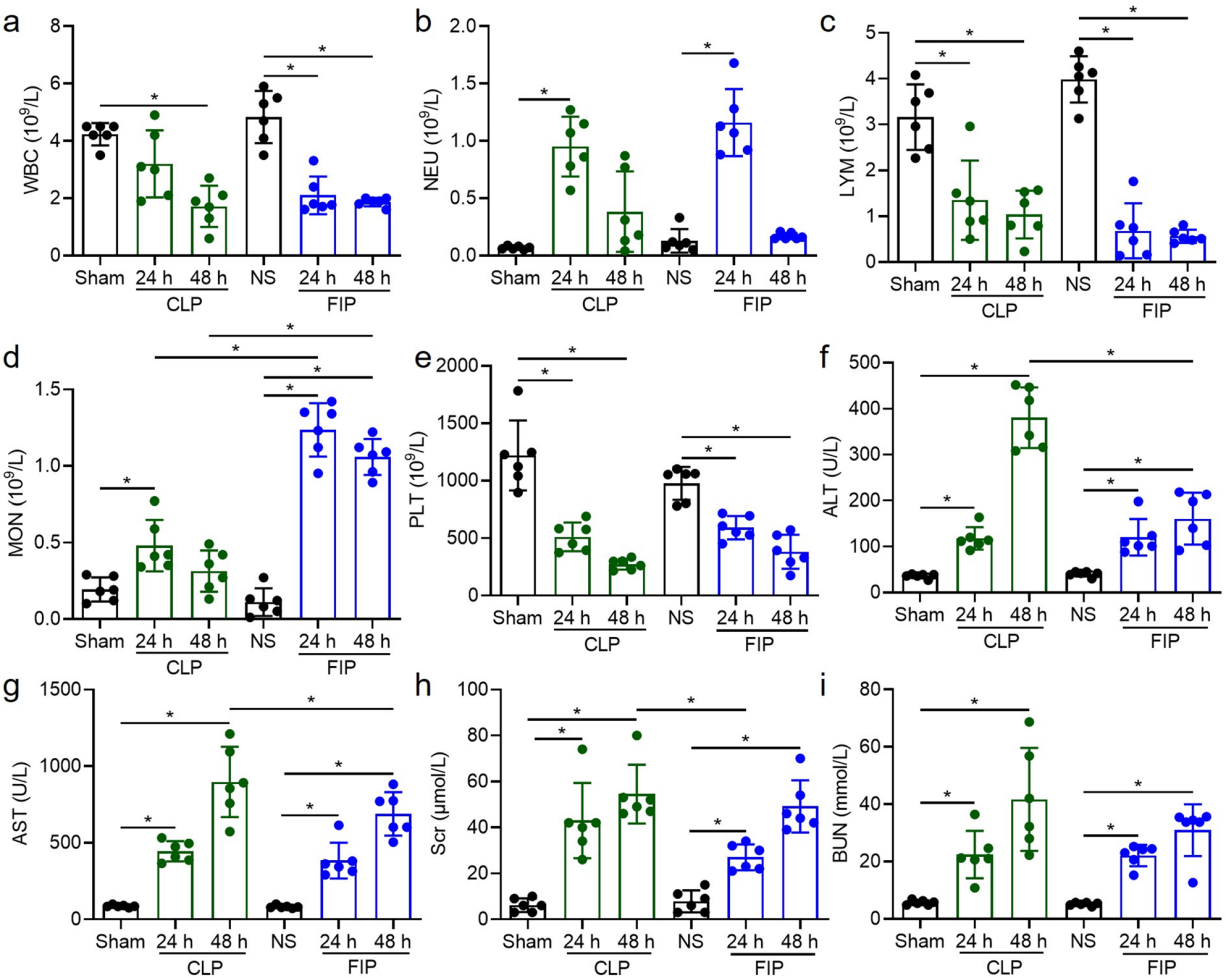
Hematological parameters and systemic biomarkers of hepatic and renal dysfunction. a-e Complete blood count analysis, including white blood cells (WBC), neutrophils (NEU), lymphocytes (LYM), monocytes (MON), and platelets (PLT). f-i Serum biochemical markers: alanine aminotransferase (ALT), aspartate aminotransferase (AST), serum creatinine (Scr), and blood urea nitrogen (BUN). Data are presented as mean ± SD with individual data points overlaid (*n* = 6). Statistical significance between the indicated groups is highlighted with brackets (^*^P < 0.05). CLP: cecal ligation and puncture; FIP: fecal intraperitoneal injection; NS: normal saline. Exact *P* values for all relevant statistical comparisons are detailed in Additional file 2

Both models significantly altered peripheral blood cell counts, including a marked reduction in WBC counts in CLP mice at 48 h *(P* < 0.05) and in FIP mice at all assessed time points *(P* < 0.05) (Fig. 4a). Significant increases in NEU counts at 24 h *(P* < 0.05), suppression of LYM counts *(P* < 0.05), and elevations in MON counts at 24 h *(P* < 0.05) were observed in both groups, with FIP mice exhibiting significantly higher MON counts than CLP mice at both 24 and 48 h *(P* < 0.05) (Fig. 4b, c, d). PLT counts were also significantly decreased in both models *(P* < 0.05) (Fig. 4e). Serum biochemical analysis revealed significant elevations in ALT, AST, BUN, and Scr in both models compared to controls *(P* < 0.05) (Fig. 4f-i). Notably, Scr was significantly lower in FIP mice than in CLP mice at 24 h *(P* < 0.05), whereas ALT and AST levels were significantly higher in the CLP group than in the FIP group by 48 h *(P* < 0.05) (Fig. 4f-i).

Taken together, these findings indicate that the optimized FIP model induces a more rapid and acute systemic inflammatory response and hematological disruption. Conversely, the CLP model leads to more progressive and sustained hepatic and renal impairment, reflecting the different pathological dynamics of a single-bolus infection versus a continuous infectious leak.

### Multi-organ histopathological injury

**Fig. 5:**
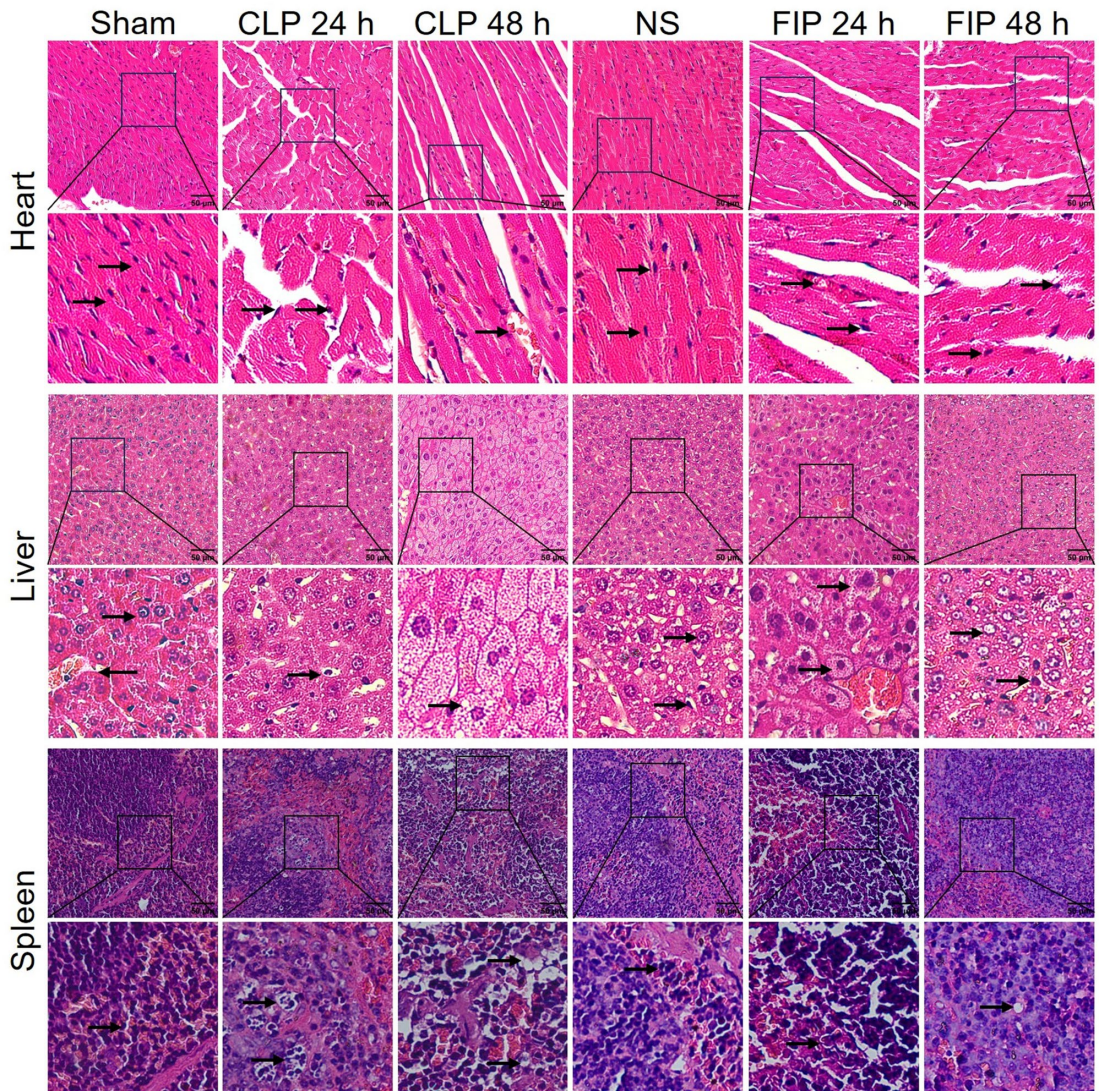
Histopathological evaluation of the heart, liver, and spleen in CLP and FIP sepsis models. Representative H&E-stained sections from Sham, CLP (24 and 48 h), NS, and FIP (24 and 48 h) groups are shown. Black arrows indicate specific morphological alterations in each tissue panel: heart, myocardial fiber disorganization and interstitial edema; liver, hepatocellular vacuolar degeneration and swelling; spleen, blurring of the red and white pulp boundaries. Scale bar = 50 μm (*n* = 6). For histological evaluation, at least 10 randomly selected high-power fields were analyzed per organ for each mouse. CLP: cecal ligation and puncture; FIP: fecal intraperitoneal injection; NS: normal saline

**Fig. 6:**
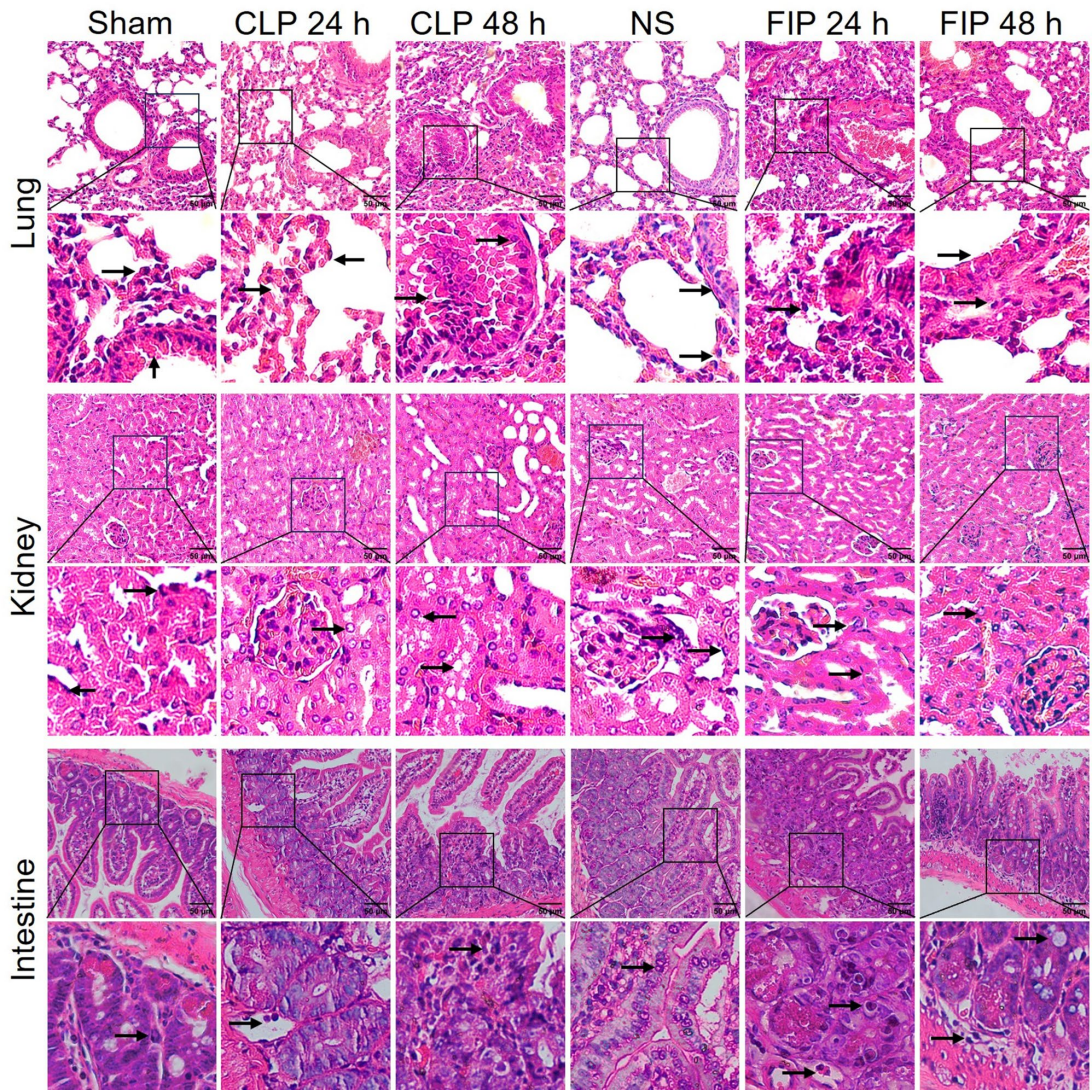
Histopathological evaluation of the lung, kidney, and intestine in CLP and FIP sepsis models. Representative H&E-stained sections from Sham, CLP (24 and 48 h), NS, and FIP (24 and 48 h) groups are shown. Black arrows indicate specific morphological alterations in each tissue panel: lung, alveolar septal thickening and inflammatory infiltration; kidney, tubular epithelial necrosis and glomerular shrinkage; intestine, mucosal sloughing and villi blunting. Scale bar = 50 μm (*n* = 6). For histological evaluation, at least 10 randomly selected high-power fields were analyzed per organ for each mouse. CLP: cecal ligation and puncture; FIP: fecal intraperitoneal injection; NS: normal saline

Histopathological evaluation of major organs, including the heart, liver, lung, kidney, and intestine, provides direct morphological evidence of systemic tissue damage. H&E staining revealed characteristic multi-organ injuries in both models (Figs. 5 and 6). In the heart, we observed myocardial fiber fragmentation and interstitial edema. In the liver, significant hepatocellular swelling and vacuolar degeneration were evident. Myocardial and hepatic injuries were more prominent during the acute phase (24 h) in the FIP group, whereas they appeared more severe and persistent at 48 h in the CLP group.

Pulmonary pathology in the FIP group was characterized by early-onset alveolar wall thickening and inflammatory infiltration at 24 h; in contrast, lung injury in the CLP group was more protracted. Renal damage, manifested by glomerular shrinkage and acute tubular necrosis, progressed over time in the CLP group and peaked at 48 h, while FIP induced substantial injury as early as 24 h. Furthermore, intestinal injury—consisting of mucosal edema, villi blunting, and epithelial necrosis—tended to be more severe and sustained in the CLP group, likely driven by the continuous leakage of infectious material from the cecum.

**Fig. 7:**
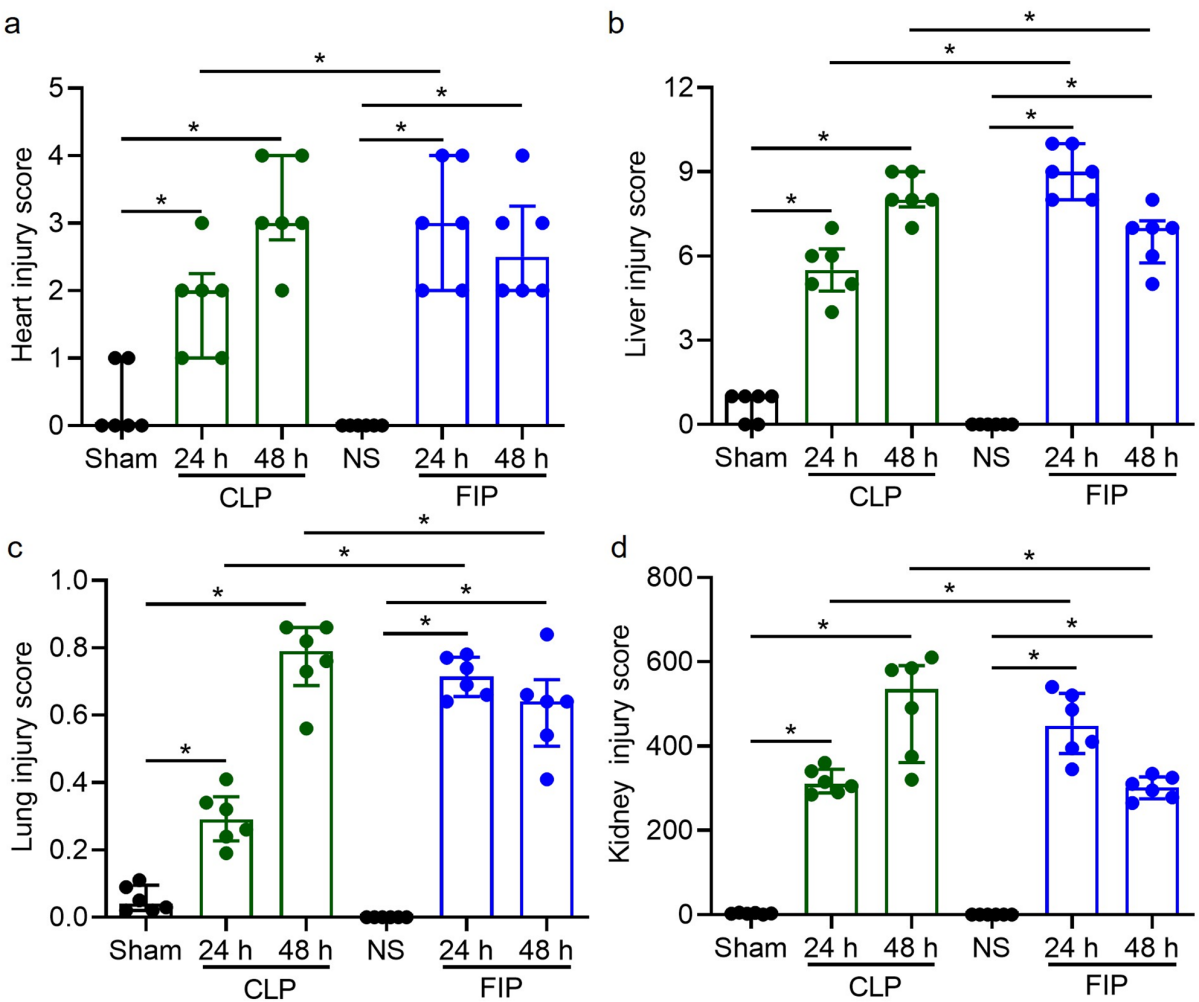
Semi-quantitative histological scores of multi-organ injury. a-d Histological injury scores for the heart (**a**), liver (**b**), lung (**c**), and kidney (**d**). Data are presented as median with interquartile range (IQR) with individual data points overlaid (*n* = 6). Statistical significance between the indicated groups is highlighted with brackets (*P* < 0.05). CLP: cecal ligation and puncture; FIP: fecal intraperitoneal injection; NS: normal saline. Exact *P* values for all relevant statistical comparisons are detailed in Additional file 2

To evaluate sepsis-induced multi-organ dysfunction syndrome (MODS), semi-quantitative histological scoring was performed on the lung, kidney, liver, and heart, allowing for a precise mapping of the distinct injury kinetics between the FIP and CLP models (Fig. 7). Consistent with the microscopic observations, both CLP and FIP models exhibited significantly higher injury scores across all evaluated organs (heart, liver, lung, and kidney) at 24 and 48 h compared to their respective sham or normal saline controls (*P* < 0.05, Fig. 7a-d). Direct comparison between the two sepsis models revealed distinct temporal dynamics of organ failure. The FIP group demonstrated a hyperacute injury phenotype, evidenced by significantly higher histopathological scores in the heart, liver, lung, and kidney at 24 h relative to the CLP group (*P* < 0.05). Conversely, as the infection progressed to 48 h, the CLP model exhibited continuous exacerbation of tissue damage. By 48 h, the injury scores for the liver, lung, and kidney in the CLP group significantly surpassed those in the FIP group (*P* < 0.05, Fig. 7b-d), aligning with the sustained infectious leak characteristic of the CLP procedure. Cardiac injury scores remained elevated but showed no significant difference between the two models at this later time point (Fig. 7a).

## Discussion

In the present study, an optimized murine model of polymicrobial sepsis was established and systematically evaluated using fecal intraperitoneal injection. This optimization was primarily driven by the inherent limitations of conventional dry fecal preparations. As explicitly highlighted in Fig. 1, the dry fecal suspension model exhibited poor reproducibility and highly inconsistent survival outcomes across different batches. Our central findings indicate that a standardized fresh fecal suspension administered at a dose of 0.7 g/kg generates key outcomes—including mortality, multi-organ dysfunction, and systemic bacterial burdens—that are broadly comparable to those produced by the conventional CLP model. Crucially, the optimized protocol demonstrates substantial advantages in terms of reproducibility, procedural simplicity, and controllability of disease severity, thereby supporting its utility as a reliable and efficient preclinical platform for sepsis research and therapeutic evaluation.

Although CLP is widely regarded as the gold standard for modeling polymicrobial sepsis, its primary limitation remains high inter-individual variability and a strong dependency on surgical technique. In the present study, mice in the CLP group exhibited pronounced disease severity. This observation aligns with the findings of Jain et al., who reported that mortality in the CLP control group reached 100% within 24 h in the absence of effective therapeutic intervention [12]. Such high lethality further underscores the stringency of the CLP model in simulating severe sepsis and assessing pharmacological efficacy. However, consistency in outcomes is often complicated by variations in the extent of ischemic necrosis in the ligated cecal segment [23]. Furthermore, variability arises from multiple factors that are difficult to standardize, such as the exact ligation position, needle gauge, number of punctures, and operator proficiency, all of which directly determine the initial infectious burden. Additionally, anatomical and local pathological differences among mice can lead to the partial occlusion of puncture sites by adjacent tissues, thereby altering the leakage dynamics of infectious material [24]. These complex local interactions collectively make the strict standardization of the CLP model challenging.

By contrast, the optimized FIP approach mitigates these sources of variability through the systematic control of the infectious source, the preparation process, and the dosing strategy. First, the immediate collection of fresh feces from donor mice maximizes the preservation of microbial viability and the community complexity, which more faithfully models polymicrobial infection while remaining ethically compatible [25]. Although direct metagenomic profiling was not performed in the current study, it is well-established that the healthy SPF murine gut microbiome is predominantly composed of Bacteroidota and Bacillota (formerly Bacteroidetes and Firmicutes), alongside crucial opportunistic pathogens such as Escherichia coli and Enterococcus species [26]. The introduction of this complex, symbiotic microbial consortium into the sterile peritoneal cavity rapidly transforms it into a highly virulent polymicrobial inoculum, successfully driving the profound systemic infection observed in our FIP model [27]. Second, the implementation of a two-step filtration workflow—utilizing a 0.5 mm mesh followed by 70 μm filtration—improves suspension homogeneity and injectability. This refinement significantly reduces the risk of syringe clogging and potential particulate-related complications, thereby increasing overall procedural reliability. Third, graded dosing experiments identified 0.7 g/kg as an optimal dose that yields moderate mortality (approximately 50%) and a severity comparable to CLP, thus providing a practical therapeutic intervention window.

Regarding disease assessment, the MSS effectively differentiates healthy from septic states and captures temporal changes in severity; however, its predictive value is intrinsically limited. Notably, a subset of mice died before reaching peak MSS, a finding consistent with multicenter reports of fecal-induced peritonitis models [28]. This likely reflects fatal physiological derangements, such as malignant arrhythmias, abrupt hypotension, or catastrophic homeostatic collapse, which can precede the overt behavioral deterioration captured by the MSS [29]. Therefore, while the MSS remains a valuable tool, it should be integrated with objective laboratory indices and, where feasible, continuous physiological monitoring to improve phenotyping accuracy in sepsis studies.

Beyond procedural differences, our data suggest that FIP and CLP model distinct sepsis trajectories. The CLP model establishes a persistent infectious focus (a necrotic nidus) with ongoing leakage, thereby mimicking sepsis driven by unresolved source control. In contrast, the FIP model delivers a single, high-load polymicrobial challenge. These divergent infection mechanisms likely explain the temporal crossover in peritoneal bacterial loads observed between the two models (Fig. 3b). We hypothesize that the immediate, massive bacterial bolus in FIP triggers an intense and rapid local recruitment of innate immune cells (e.g., neutrophils and macrophages) into the peritoneal cavity, which may facilitate a relatively more effective early bacterial clearance. Conversely, the continuous pathogen release and local tissue ischemia in CLP constantly overwhelm localized immune defenses, driving a progressively ascending bacterial burden over time. Although we did not directly quantify peritoneal leukocyte populations to confirm these cellular dynamics in the current study, these distinct mechanistic pathways are highly consistent with the observed downstream pathologies. Indeed, CLP produces a more protracted clinical course characterized by sustained or worsening organ injury at later time points [30]. Meanwhile, the FIP model leads to earlier bacteremia peaks and rapid multi-organ injury within 24 h. Notably, the FIP group exhibited lung edema and inflammatory infiltration as early as 24 h, whereas CLP-induced damage was more protracted. This rapid onset aligns with the biological vulnerability of the lung as the most critical organ during sepsis [31]. Our observation of sustained high bacterial loads contrasts with reports of declining counts in similar fresh-fecal models [32]. This discrepancy is likely attributable to differences in therapeutic intervention and animal species. Tallósy et al. utilized a rat model and administered fluid resuscitation and analgesics following induction, interventions known to bolster hemodynamic stability and facilitate host immune clearance of pathogens. In contrast, our study employed a mouse model without fluid resuscitation to establish a baseline of unmitigated sepsis. Consequently, the compromised host defense in our model likely failed to limit bacterial proliferation, resulting in persistently elevated peritoneal bacterial burdens typical of fulminant, untreated sepsis. Accordingly, CLP and FIP should be viewed as complementary rather than interchangeable models: FIP may be preferable for studying early inflammatory storms and rapid innate immune activation (supported by the acute hematological shifts in Fig. 4). Particularly, the FIP model was characterized by a rapid and profound leukopenia (Fig. 4a). Biologically, this sharp decline in circulating white blood cells during fulminant polymicrobial peritonitis is primarily attributed to the massive margination and expedited migration of leukocytes into the primary site of infection (the peritoneal cavity), coupled with sepsis-induced leukocyte apoptosis and the rapid exhaustion of the circulating immune cell pool [33]. On the other hand, CLP is more suitable for investigating sustained infection, sepsis-associated immunosuppression, prolonged organ dysfunction, or secondary infections (consistent with the progressive hepatic and renal damage observed at 48 h in Figs. 4 and 6).

Multi-dimensional evaluation further confirmed that the optimized FIP model successfully reproduces the core features of sepsis, including systemic bacterial dissemination, organ dysfunction (indicated by elevated ALT, AST [34], BUN, and Scr [35]), and multi-organ histopathological injury. Furthermore, the newly incorporated semi-quantitative histological scoring (Fig. 7) provides robust morphological evidence for the divergent pathophysiological trajectories observed between the two models. The hyperacute spike in organ injury scores in the FIP group at 24 h, particularly within hepatic and pulmonary tissues, aligns with the massive and immediate systemic challenge of PAMPs and DAMPs following bolus injection. Conversely, the progressive exacerbation of hepatic and renal damage through 48 h in the CLP model reflects the clinical reality of an unresolved, protracted intra-abdominal infectious source. These semi-quantitative data effectively bridge the gap between qualitative microscopic observations and systemic biochemical markers, offering a more comprehensive validation of model-specific organ dysfunction.

However, several limitations should be acknowledged. First, to isolate the variability of the induction method itself, this study purposefully excluded therapeutic interventions (e.g., fluid resuscitation, antibiotics). While necessary for standardization, this absence of circulatory support means the model represents an unmitigated, fulminant sepsis trajectory, differing from the managed clinical course seen in human patients. Second, although our optimized FIP model yielded consistent pathophysiological responses, we did not quantitatively characterize the baseline bacterial load (CFU/mL) or the specific microbial composition of the fecal suspensions across different batches. Without direct microbiological profiling, potential batch-to-batch variation cannot be entirely ruled out. We sought to minimize this risk through a highly standardized preparation protocol, which included pooling donor samples, using fixed collection times, and strictly controlling the temperature during preparation. Third, while we comprehensively evaluated the macroscopic and functional outcomes of the models, we did not profile the intermediate immunological kinetics, such as serum inflammatory cytokines (e.g., TNF-α and IL-6). Finally, the current study was limited to young male mice. Future research should validate this protocol in aged and female cohorts, incorporate metagenomic sequencing and CFU analysis to better characterize the specific ‘sepsis-inducing’ microbiome components, and meticulously investigate the cytokine storm dynamics driving the rapid pathogenesis in this model.

## Conclusions

Collectively, this study successfully establishes and validates a highly standardized and reproducible fresh FIP murine model of polymicrobial sepsis. Characterized by operational simplicity and tunable severity, this model offers a robust and cost-effective platform for investigating the complex pathophysiology of moderately severe sepsis. Furthermore, by delineating the fundamental temporal and pathological divergence between the FIP and CLP models, these findings provide critical practical guidance for selecting the most appropriate experimental framework based on specific research objectives.

## Supplementary Information

Below is the link to the electronic supplementary material.

Supplementary Material 1: This table details the clinical parameters and the scoring system utilized to evaluate disease severity in the murine sepsis models.

Supplementary Material 2: This table provides the exact P values for all relevant inter-group statistical comparisons presented in Figures 2, 3, 4, and 7.

## Data Availability

The datasets used and/or analysed during the current study are available from the corresponding author on reasonable request.

## Abbreviations

ALT: Alanine aminotransferase
AST: Aspartate aminotransferase
BUN: Blood urea nitrogen
CFU: Colony forming unit
CLP: Cecal ligation and puncture
FIP: Fecal intraperitoneal injection
LPS: Lipopolysaccharide
LYM: Lymphocyte
MON: Monocyte
MSS: Murine Sepsis Score
NEU: Neutrophil
PCF: Peritoneal cavity fluid
PLT: Platelet
Scr: Serum creatinine
WBC: White blood cell
