# Supplementary material for: Optimization of a fresh fecal intraperitoneal injection sepsis model and its divergent dynamics from cecal ligation and puncture in mice
Source: Lab Anim Res. 2026 May 28;42:20. doi: 10.1186/s42826-026-00282-w (PMC13220502; doi:10.1186/s42826-026-00282-w)
Supplement: Supplementary file 1 — Supplementary Material 1: This table details the clinical parameters and the scoring system utilized to evaluate disease severity in the murine sepsis models. [file 42826_2026_282_MOESM1_ESM.docx]

**Additional file 1: Murine Sepsis Score (MSS) assessment criteria.**

| **Variable** | **Description** | **Score** |
| --- | --- | --- |
| Appearance | Coat is smooth | 0 |
|  | Patches of hair piloerected | 1 |
|  | Majority of back is piloerected | 2 |
|  | Piloerection may or may not be present, mouse appears “puffy” | 3 |
|  | Piloerection may or may not be present, mouse appears emaciated | 4 |
| Level of consciousness | Mouse is active | 0 |
|  | Mouse is active but avoids standing upright | 1 |
|  | Mouse activity is noticeably slowed. The mouse is still ambulant. | 2 |
|  | Activity is impaired. Mouse only moves when provoked, movements have a tremor | 3 |
|  | Activity severely impaired. Mouse remains stationary when provoked, with possible tremor | 4 |
| Activity | Normal amount of activity. Mouse is any of: eating, drinking, climbing, running, fighting | 0 |
|  | Slightly suppressed activity. Mouse is moving around bottom of cage | 1 |
|  | Suppressed activity. Mouse is stationary with occasional investigative movements | 2 |
|  | No activity. Mouse is stationary | 3 |
|  | No activity. Mouse experiencing tremors, particularly in the hind legs | 4 |
| Response to stimulus | Mouse responds immediately to auditory stimulus or touch | 0 |
|  | Slow or no response to auditory stimulus; strong response to touch (moves to escape | 1 |
|  | No response to auditory stimulus; moderate response to touch (moves a few steps | 2 |
|  | No response to auditory stimulus; mild response to touch (no locomotion) | 3 |
|  | No response to auditory stimulus. Little or no response to touch. Cannot right itself if pushed over | 4 |
| Eyes | Open | 0 |
|  | Eyes not fully open, possibly with secretions | 1 |
|  | Eyes at least half closed, possibly with secretions | 2 |
|  | Eyes half closed or more, possibly with secretions | 3 |
|  | Eyes closed or milky | 4 |

| **Variable** | **Description** | **Score** |
| --- | --- | --- |
| Respiration rate | Normal, rapid mouse respiration | 0 |
|  | Slightly decreased respiration (rate not quantifiable by eye) | 1 |
|  | Moderately reduced respiration (rate at the upper range of quantifying by eye) | 2 |
|  | Severely reduced respiration (rate easily countable by eye, 0.5 s between breaths) | 3 |
|  | Extremely reduced respiration (> 1 s between breaths) | 4 |
| Respiration quality | Normal | 0 |
|  | Brief periods of laboured breathing | 1 |
|  | Laboured, no gasping | 2 |
|  | Laboured with intermittent gasps | 3 |
|  | Gasping | 4 |
