# Supplementary material for: Optimization of a fresh fecal intraperitoneal injection sepsis model and its divergent dynamics from cecal ligation and puncture in mice
Source: Lab Anim Res. 2026 May 28;42:20. doi: 10.1186/s42826-026-00282-w (PMC13220502; doi:10.1186/s42826-026-00282-w)
Supplement: Supplementary file 2 — Supplementary Material 2: This table provides the exact P values for all relevant inter-group statistical comparisons presented in Figures 2, 3, 4, and 7. [file 42826_2026_282_MOESM2_ESM.docx]

**Additional file 2: Exact *P* values for statistical comparisons.**

| **Figure Panel** | **Comparison** | **Exact *P* value** | **Significance** |
| --- | --- | --- | --- |
| Fig. 2b | CLP 12-h vs. FIP 12-h | 0.002 | * |
| Fig. 2b | CLP 24-h vs. FIP 24-h | 0.002 | * |
| Fig. 2b | CLP 36-h vs. FIP 36-h | 0.002 | * |
| Fig. 2b | CLP 48-h vs. FIP 48-h | 0.378 | ns |
| Fig. 2b | CLP 60-h vs. FIP 60-h | 0.931 | ns |
| Fig. 2b | CLP 72-h vs. FIP 72-h | 0.748 | ns |
| Fig. 2c | CLP 24-h vs. Sham | 0.041 | * |
| Fig. 2c | CLP 48-h vs. Sham | 0.014 | * |
| Fig. 2c | FIP 24-h vs. NS | 0.001 | * |
| Fig. 2c | FIP 48-h vs. NS | < 0.001 | * |
| Fig. 3a | CLP 24-h vs. Sham | < 0.001 | * |
| Fig. 3a | CLP 48-h vs. Sham | 0.010 | * |
| Fig. 3a | FIP 24-h vs. NS | < 0.001 | * |
| Fig. 3a | FIP 48-h vs. NS | < 0.001 | * |
| Fig. 3a | FIP 24-h vs. CLP 24-h | 0.037 | * |
| Fig. 3a | FIP 48-h vs. CLP 48-h | 0.008 | * |
| Fig. 3b | CLP 24-h vs. Sham | 0.007 | * |
| Fig. 3b | CLP 48-h vs. Sham | < 0.001 | * |
| Fig. 3b | FIP 24-h vs. NS | < 0.001 | * |
| Fig. 3b | FIP 48-h vs. NS | < 0.001 | * |
| Fig. 3b | FIP 24-h vs. CLP 24-h | 0.017 | * |
| Fig. 3b | FIP 48-h vs. CLP 48-h | 0.002 | * |
| Fig. 4a | CLP 24-h vs. Sham | 0.086 | ns |
| Fig. 4a | CLP 48-h vs. Sham | < 0.001 | * |
| Fig. 4a | FIP 24-h vs. NS | < 0.001 | * |
| Fig. 4a | FIP 48-h vs. NS | < 0.001 | * |
| Fig. 4a | FIP 24-h vs. CLP 24-h | 0.061 | ns |
| Fig. 4a | FIP 48-h vs. CLP 48-h | 0.996 | ns |
| Fig. 4b | CLP 24-h vs. Sham | < 0.001 | * |
| Fig. 4b | CLP 48-h vs. Sham | 0.072 | ns |
| Fig. 4b | FIP 24-h vs. NS | < 0.001 | * |
| Fig. 4b | FIP 48-h vs. NS | 0.997 | ns |
| Fig. 4b | FIP 24-h vs. CLP 24-h | 0.344 | ns |
| Fig. 4b | FIP 48-h vs. CLP 48-h | 0.330 | ns |
| Fig. 4c | CLP 24-h vs. Sham | < 0.001 | * |
| Fig. 4c | CLP 48-h vs. Sham | < 0.001 | * |
| Fig. 4c | FIP 24-h vs. NS | < 0.001 | * |
| Fig. 4c | FIP 48-h vs. NS | < 0.001 | * |
| Fig. 4c | FIP 24-h vs. CLP 24-h | 0.219 | ns |
| Fig. 4c | FIP 48-h vs. CLP 48-h | 0.527 | ns |
| Fig. 4d | CLP 24-h vs. Sham | 0.003 | * |
| Fig. 4d | CLP 48-h vs. Sham | 0.395 | ns |
| Fig. 4d | FIP 24-h vs. NS | < 0.001 | * |
| Fig. 4d | FIP 48-h vs. NS | < 0.001 | * |
| Fig. 4d | FIP 24-h vs. CLP 24-h | < 0.001 | * |
| Fig. 4d | FIP 48-h vs. CLP 48-h | < 0.001 | * |
| Fig. 4e | CLP 24-h vs. Sham | < 0.001 | * |

| **Figure Panel** | **Comparison** | **Exact *P* value** | **Significance** |
| --- | --- | --- | --- |
| Fig. 4e | CLP 48-h vs. Sham | < 0.001 | * |
| Fig. 4e | FIP 24-h vs. NS | 0.002 | * |
| Fig. 4e | FIP 48-h vs. NS | < 0.001 | * |
| Fig. 4e | FIP 24-h vs. CLP 24-h | 0.867 | ns |
| Fig. 4e | FIP 48-h vs. CLP 48-h | 0.678 | ns |
| Fig. 4f | CLP 24-h vs. Sham | 0.006 | * |
| Fig. 4f | CLP 48-h vs. Sham | < 0.001 | * |
| Fig. 4f | FIP 24-h vs. NS | 0.008 | * |
| Fig. 4f | FIP 48-h vs. NS | < 0.001 | * |
| Fig. 4f | FIP 24-h vs. CLP 24-h | 0.999 | ns |
| Fig. 4f | FIP 48-h vs. CLP 48-h | < 0.001 | * |
| Fig. 4g | CLP 24-h vs. Sham | 0.001 | * |
| Fig. 4g | CLP 48-h vs. Sham | < 0.001 | * |
| Fig. 4g | FIP 24-h vs. NS | 0.001 | * |
| Fig. 4g | FIP 48-h vs. NS | < 0.001 | * |
| Fig. 4g | FIP 24-h vs. CLP 24-h | 0.867 | ns |
| Fig. 4g | FIP 48-h vs. CLP 48-h | 0.027 | * |
| Fig. 4h | CLP 24-h vs. Sham | < 0.001 | * |
| Fig. 4h | CLP 48-h vs. Sham | < 0.001 | * |
| Fig. 4h | FIP 24-h vs. NS | 0.012 | * |
| Fig. 4h | FIP 48-h vs. NS | < 0.001 | * |
| Fig. 4h | FIP 24-h vs. CLP 24-h | 0.044 | * |
| Fig. 4h | FIP 48-h vs. CLP 48-h | 0.832 | ns |
| Fig. 4i | CLP 24-h vs. Sham | 0.013 | * |
| Fig. 4i | CLP 48-h vs. Sham | < 0.001 | * |
| Fig. 4i | FIP 24-h vs. NS | 0.012 | * |
| Fig. 4i | FIP 48-h vs. NS | < 0.001 | * |
| Fig. 4i | FIP 24-h vs. CLP 24-h | > 0.999 | ns |
| Fig. 4i | FIP 48-h vs. CLP 48-h | 0.172 | ns |
| Fig. 7a | CLP 24-h vs. Sham | 0.003 | * |
| Fig. 7a | CLP 48-h vs. Sham | < 0.001 | * |
| Fig. 7a | FIP 24-h vs. NS | < 0.001 | * |
| Fig. 7a | FIP 48-h vs. NS | < 0.001 | * |
| Fig. 7a | FIP 24-h vs. CLP 24-h | 0.027 | * |
| Fig. 7a | FIP 48-h vs. CLP 48-h | 0.601 | ns |
| Fig. 7b | CLP 24-h vs. Sham | < 0.001 | * |
| Fig. 7b | CLP 48-h vs. Sham | < 0.001 | * |
| Fig. 7b | FIP 24-h vs. NS | < 0.001 | * |
| Fig. 7b | FIP 48-h vs. NS | < 0.001 | * |
| Fig. 7b | FIP 24-h vs. CLP 24-h | < 0.001 | * |
| Fig. 7b | FIP 48-h vs. CLP 48-h | 0.012 | * |
| Fig. 7c | CLP 24-h vs. Sham | < 0.001 | * |
| Fig. 7c | CLP 48-h vs. Sham | < 0.001 | * |
| Fig. 7c | FIP 24-h vs. NS | < 0.001 | * |
| Fig. 7c | FIP 48-h vs. NS | < 0.001 | * |
| Fig. 7c | FIP 24-h vs. CLP 24-h | < 0.001 | * |
| Fig. 7c | FIP 48-h vs. CLP 48-h | 0.029 | * |

| **Figure Panel** | **Comparison** | **Exact *P* value** | **Significance** |
| --- | --- | --- | --- |
| Fig. 7d | CLP 24-h vs. Sham | < 0.001 | * |
| Fig. 7d | CLP 48-h vs. Sham | < 0.001 | * |
| Fig. 7d | FIP 24-h vs. NS | < 0.001 | * |
| Fig. 7d | FIP 48-h vs. NS | < 0.001 | * |
| Fig. 7d | FIP 24-h vs. CLP 24-h | 0.003 | * |
| Fig. 7d | FIP 48-h vs. CLP 48-h | < 0.001 | * |

Abbreviations: ns, not significant. * Indicates statistical significance (*P* < 0.05).
